# Magnetic Dynamics and Elongated Coherence of a High‐Spin Mn(II) Qubit Doped Into a Metal‐Organic Framework

**DOI:** 10.1002/chem.202502971

**Published:** 2025-12-17

**Authors:** Shraddha Gupta, Masanori Wakizaka, Takeshi Yamane, Hisaaki Tanaka, Ryuta Ishikawa, Shinya Takaishi, Kazunobu Sato, Masahiro Yamashita

**Affiliations:** ^1^ Department of Chemistry, Graduate School of Science Tohoku University Sendai Japan; ^2^ Department of Applied Chemistry and Bioscience, Faculty of Science and Technology Chitose Institute of Science and Technology Chitose Japan; ^3^ Department of Chemistry, Graduate School of Science Osaka Metropolitan University Sumiyoshi‐Ku Osaka Japan; ^4^ Department of Chemistry, Faculty of Science Fukuoka University Jonan‐Ku Fukuoka Japan; ^5^ School of Chemical Science and Engineering Tongji University Shanghai PR China

**Keywords:** electron spin resonance, magnetism, manganese(II), metal‐organic framework, spin qubit

## Abstract

Spin qubits are among the most promising candidates for quantum information processing and sensing technologies. Their potential to function even at elevated temperatures makes them particularly attractive for future devices. However, while extensive studies have been carried out on *S* = 1/2 systems, high‐spin complexes remain much less explored as spin qubit platforms. In this study, we prepared a Zn(II)‐based MOF, [CH_6_N_3_][Zn(HCOO)_3_], doped with trace amounts of Mn(II) ions (*S* = 5/2, 0.2, and 0.02 mol%). Magnetic measurements under static fields revealed slow relaxation phenomena dominated by direct and Raman‐like processes. Importantly, Q‐band pulsed ESR confirmed quantum coherence between *M*_S_ = ±1/2 sublevels, achieving phase memory times (*T*_2_) up to 5.4 µs at 10 K, which is significantly longer than those reported in other Mn(II)‐based systems. Rabi nutation experiments verified coherent spin control and multilevel transitions, while Wigner matrix analysis revealed reorientation of the nuclear quantization axis during spin flips. Notably, coherence persisted above 150 K, attributed to the stabilization provided in the MOF's hydrogen‐bonded lattice. This work represents the first demonstration of high‐spin Mn(II) qubits with measurable coherence at elevated temperatures, underscoring MOFs as versatile and tunable platforms for advancing quantum materials and molecular spin‐based technologies.

## Graphical abstract

This study demonstrates that dilute Mn(II) (∼0.02 mol%) doped into the Zn(II)‐MOF [CH_6_N_3_][Zn(HCOO)_3_] act as high‐spin (*S* = 5/2) spin qubits, achieving microsecond phase memory times above 50 K—the first report for Mn(II)‐based systems. Robust hydrogen‐bonding networks suppress relaxation and stabilize the lattice, extending *T*_2_. Resonance between *M*_S_ = ±1/2 and quantization axis tilting reveal tunable spin dynamics, highlighting MOFs as promising platforms for high‐spin molecular qubits.

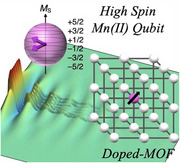

## 1 Introduction

Spin qubits operate based on the quantum superposition between two spin sublevels, such as up‐spin and down‐spin states [1, 2, 3]. Because they have the potential to function even at room temperature [4], spin qubits are considered promising candidates for applications in both quantum computing and quantum sensing [5, 6, 7]. From the perspective of chemical and physical stability, metal complexes have been actively explored as spin qubit platforms alongside nitrogen‐vacancy centers in diamond [8, 9, 10]. Previous studies on metal complex‐based spin qubits have mainly focused on metal ions with an effective spin of *S* = 1/2 such as V(IV) [11, 12, 13, 14], low‐spin Fe(III) [15, 16], low‐spin Co(II) [17], low‐spin Ni(III) [18, 19], Cu(II) [4, 20, 21, 22], Mo(V) [23], W(V) [23], Ru(III) [10], and Os(III) [10], due to their ability to maintain stable spin coherence between the *M*_S_ = ±1/2 sublevels. In contrast, high‐spin metal complexes as spin qubits have been less extensively reported and remain largely unexplored.

Achieving long‐lived spin coherence, commonly quantified as the phase memory time (*T*_m_) or spin–spin relaxation time (*T*_2_), is requires effective suppression of decoherence mechanisms. These include spin–lattice relaxation time (*T*_1_) and other magnetic relaxation time (τ), which can be mitigated through strategies such as magnetic dilution or chemical engineering of the coordination environment. Among the various candidate materials, metal–organic frameworks (MOFs) are especially attractive due to their high structural tunability, modular composition, and capacity for controlled doping of spin centers [24, 25, 26, 27]. Recent studies have reported coordination compounds and MOFs with slow magnetic relaxation, extended *T*_1_ times, and measurable quantum coherence across various structural dimensionalities, including 1D chains [28, 29, 30], 2D sheets [31], and 3D networks [6, 14, 16, 17]. Notably, hybrid perovskite‐type MOFs of the general formula {[A][Zn^II^(HCOO)_3_]}_n_ or [A][Zn^II^(HCOO)_3_] (where A is an organic cation) [32, 33] have been widely used as diamagnetic host lattices for introducing paramagnetic dopants such as high‐spin Mn(II) [34, 35, 36], high‐spin Co(II) [37], and Cu(II) [34, 36, 38]. These doped systems have demonstrated promising magnetic relaxation behavior and, in some cases, measurable spin coherence. For instance, Cu(II)‐doped MOFs with an *S* = 1/2 configuration have achieved *T*_2_ values in the microsecond range at cryogenic temperatures below 20 K [38]. In contrast, Mn(II)‐doped MOFs containing [CH_7_N_2_]^+^ [34], [C_2_H_8_N_2_]^+^ [35], and [C_4_H_14_N_2_]^+^ [36], cations have been reported to undergo phase transitions and fail to exhibit microsecond‐scale *T*_2_ even at low temperatures. Therefore, achieving microsecond‐range *T*_2_ in high‐spin systems presents both a challenge and an opportunity, and has yet to be realized. Moreover, multiple hydrogen‐bonding networks have been reported to suppress spin–lattice relaxation, offering a promising strategy for designing spin qubit systems with prolonged relaxation times [39].

**FIGURE 1:**
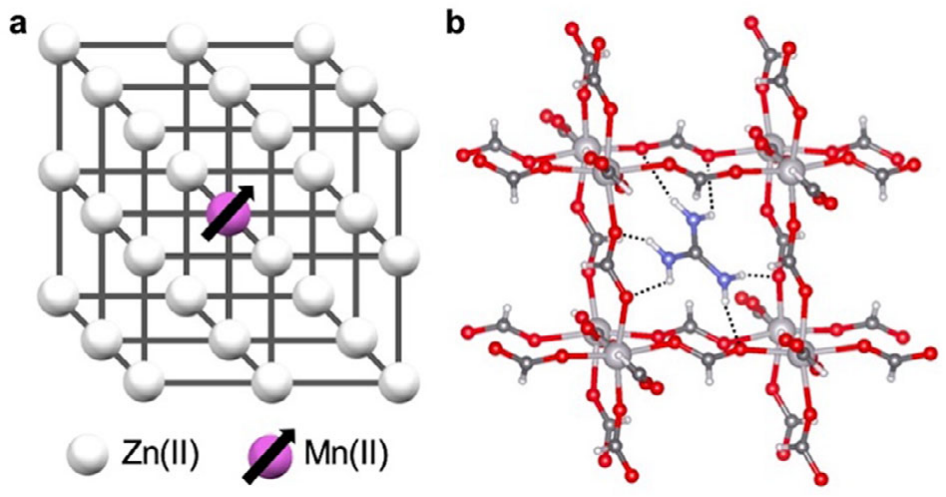
(a) Conceptual illustration of the Mn(II)‐doped Zn(II)‐based MOF. (b) Crystal structure of the host framework studied in this work, [CH_6_N_3_][Zn^II^(HCOO)3] [40].

In this study, we synthesize a Mn(II)‐doped Zn(II)‐based MOF incorporating high‐spin Mn(II) centers (*S* = 5/2) using [CH_6_N_3_][Zn^II^(HCOO)_3_] as the host framework. The MOF lattice contains guanidinium cations, which form multiple N─H···O hydrogen bonds with the formate‐based framework (Figure 1) [40]. These interactions are expected to contribute to the suppression of spin–lattice relaxation processes by reducing local vibrational coupling and phase transitions. The magnetic behavior and spin qubit properties of the embedded high‐spin Mn(II) centers are investigated and discussed in detail.

## 2 Results and Discussion

### 2.1 Synthesis and Magnetic Characterization and Relaxation

**FIGURE 2:**
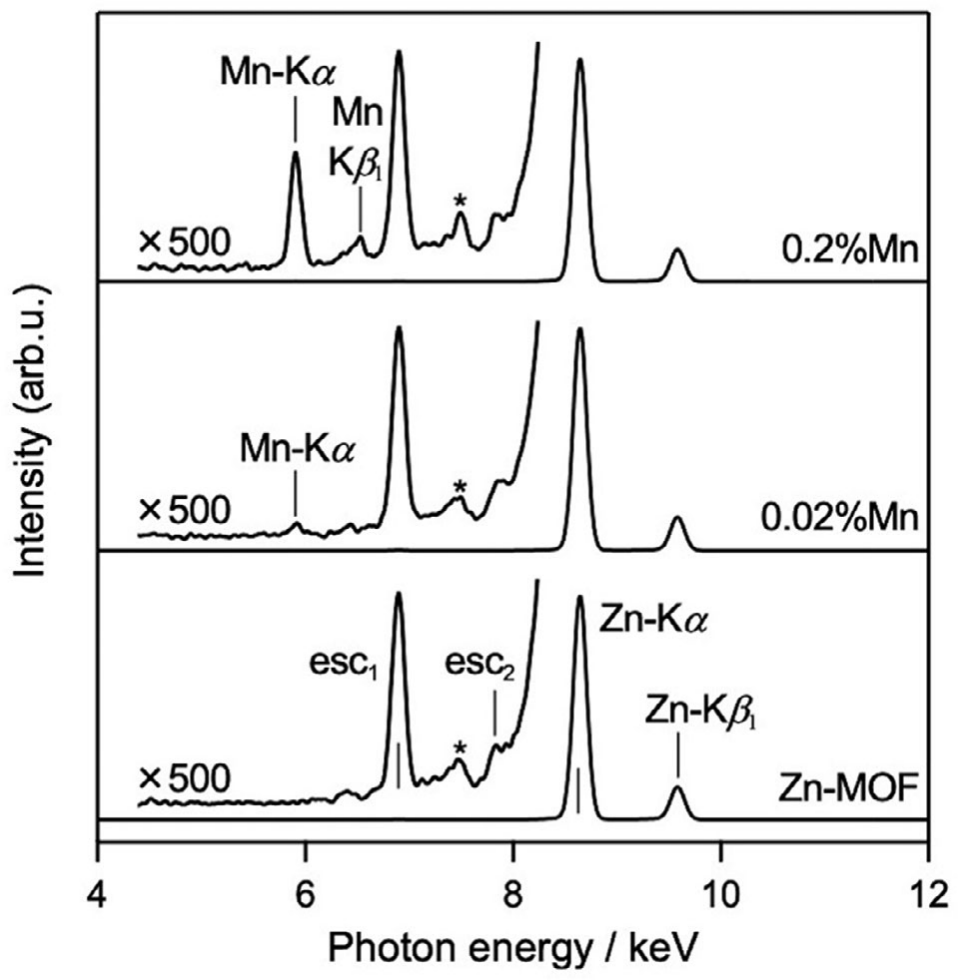
XRF spectra of Mn(II)‐doped Zn(II)‐MOFs with intended Mn loading levels of 0.2% and 0.02%, along with the pristine Zn(II)‐MOF. Insets show magnified regions around 4–8 keV (×500). The peaks labeled esc_1_ and esc_2_ correspond to the escape peaks of Zn Kα and Zn Kβ_1_, respectively. The peak marked with an asterisk (*) originates from the instrument.

Mn(II)‐doped Zn(II)‐MOFs, [CH_6_N_3_][Mn^II^_x_Zn^II^_1–x_(HCOO)_3_], were synthesized by mixing methanolic solutions of formic acid with [CH_6_N_3_]Cl and ZnCl_2_, followed by the addition of MnCl_2_ at 0.2 or 0.02 molar equivalents. The reactions proceeded almost quantitatively, with yields close to 100%. Powder X‐ray diffraction (PXRD) patterns of the Mn(II)‐doped Zn(II)‐MOFs revealed an isomorphous structure to the pristine Zn(II)‐MOF, [CH_6_N_3_][Zn^II^(HCOO)_3_] (Figure S1) [40]. X‐ray fluorescence (XRF) spectra confirmed the presence of Mn, showing characteristic peaks at 5.9 keV (Mn Kα) and 6.5 keV (Mn Kβ_1_), along with Zn matrix peaks at 8.6 keV (Zn Kα) and 9.6 keV (Zn Kβ_1_) (Figure 2). The XRF analysis using the fundamental parameter method indicated Mn‐to‐Zn molar ratios of 0.19% and 0.015% for the respective doping levels, which closely match the intended loading amounts of 0.2% and 0.02%. Based on the random doping model [38], the average distances between the doped Mn(II) sites are estimated to be ∼50 nm at 0.2% doping and ∼500 nm at 0.02% doping, respectively. The presence of Mn(II) sites in the doped MOFs was further verified by X‐band electron spin resonance (ESR) spectroscopy, which exhibited hyperfine splitting characteristic of the ^55^Mn nucleus (*I* = 5/2) (Figure S2). Additional Q‐band ESR studies using pulsed techniques were also conducted (*vide infra*). Furthermore, quantum chemical calculations on a model complex, [Mn(HCOO)_6_]^4−^, predicted nearly isotropic *g*‐values of approximately 2.00 with minimal zero‐field splitting (ZFS) (Table S1 and Figure S3), in good agreement with the experimental magnetic and ESR data for the Mn(II)‐doped MOFs discussed below.

**FIGURE 3:**
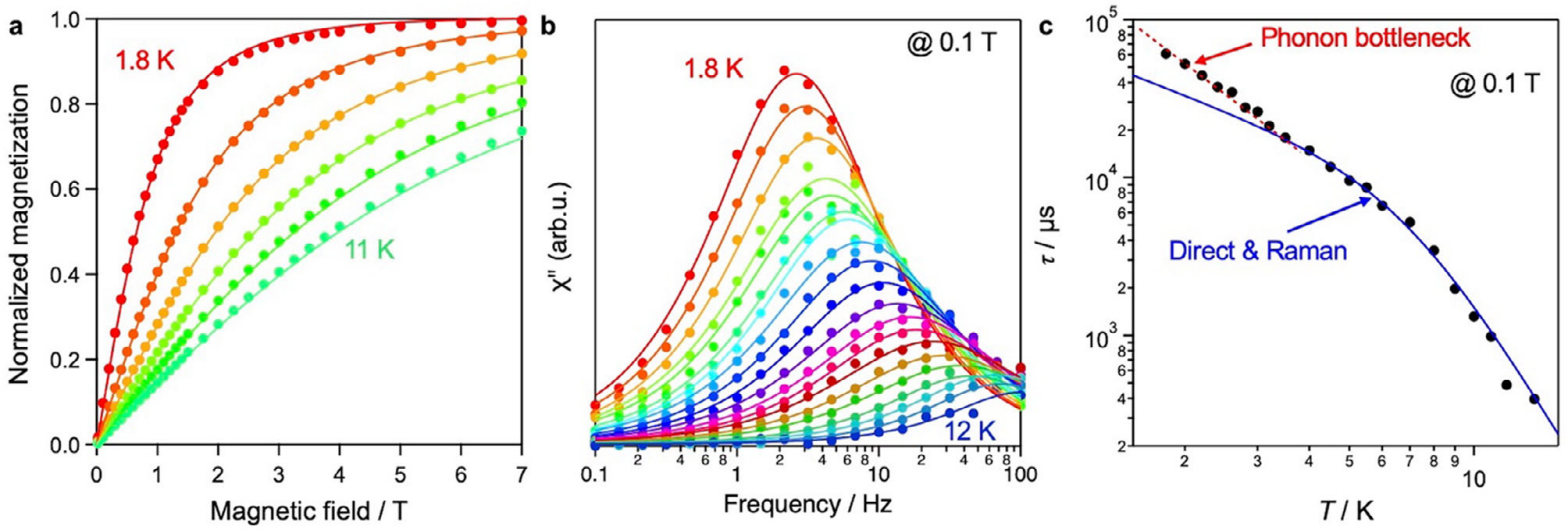
Magnetism for the 0.2% Mn(II)‐doped Zn(II)‐MOF. (a) Magnetization–field curves measured at 1.8, 3.6, 5.4, 7.2, 9.0, and 11 K, normalized to the saturation magnetization at 1.8 K. (b) Out‐of‐phase component of the AC susceptibility (χ″) measured 1.8, 2.0, 2.2, 2.4, 2.6, 2.8, 3.0, 3.2, 3.5, 4.0, 4.5, 5.0, 5.5, 6.0, 7.0, 8.0, 9.0, 10, 11, and 12 K over a frequency range of 0.1–100 Hz under a static magnetic field of 0.1 T. (c) Log‐scale plot of magnetic relaxation time (τ) as a function of temperature (*T*). Solid curves in (a) represent simulated data using parameters *g*_x_ = *g*_y_ = *g*_z_ = 2.00 and *D* = 0. Solid curves in (b) represent fits using a Debye Relaxation. Solid blue and red curves in (c) correspond to fits using the direct and Raman relaxation processes and the phonon bottleneck model, respectively.

Figure 3a shows the magnetization–field curves of the 0.2% Mn(II)‐doped Zn(II)‐MOF. The corresponding data for 0.02% doping could not be obtained due to the limited measurement sensitivity and diamagnetism. The magnetization curve at 1.8 K approaches saturation around an applied magnetic field of 7 T. The temperature dependence of these curves closely matches simulations based on a discrete isotropic single‐ion spin center with *S* = 5/2, assuming *g*_x_ = *g*_y_ = *g*_z_ = 2.00 and ZFS parameter *D* = 0 using the PHI program [41], without magnetic interactions. These results indicate that Mn(II) ions are well‐dispersed within the Zn(II)‐MOF matrix and can be regarded as nearly isotropic spin centers. The saturation magnetization per Mn atom is approximately 5 μB at 1.8 K (Figure S4), consistent with an *S* = 5/2 system without spin–orbit coupling. Owing to unavoidable uncertainties in the diamagnetic correction of magnetic measurements, more accurate magnetic parameters were determined by Q‐band ESR (vide infra). The 0.2% Mn(II)‐doped Zn(II)‐MOF does not exhibit any magnetic relaxation signals under zero static magnetic field in alternating current (AC) susceptibility measurements. However, it does show magnetic relaxation when a static magnetic field is applied (Figure S5). The temperature dependence of the AC susceptibility under a 0.1 T field is presented in Figures 3a and S6. The relaxation peaks observed in the out‐of‐phase (χ") component were analyzed using the Debye relaxation model (Equation 1) [42].

(1) $$\chi \prime \prime =\left(\chi_{T}-\chi_{S}\right)\frac{{\left(2\pi f\tau \right)}^{1-\alpha }\cos\left(\pi \alpha /2\right)}{1+2{\left(2\pi f\tau \right)}^{1-\alpha }\sin\left(\pi \alpha /2\right)+{\left(2\pi f\tau \right)}^{2-2\alpha }}$$

Here, χ_T_, χ_S_, *f*, τ, and α represent the isothermal susceptibility, adiabatic susceptibility, AC frequency, magnetic relaxation time, and dispersion coefficient, respectively (Tables S2). Magnetic relaxation occurs between the spin‐up and spin‐down sublevels arising from Zeeman splitting. Unlike ESR, this process is driven by changes in thermal population and exhibits no selectivity [22]. The magnetic relaxation time at each temperature was obtained by fitting the AC susceptibility data. Furthermore, the temperature dependence of τ was analyzed using a combination of direct and Raman‐like relaxation processes, as shown in Figure 3c and described by Equations (2), (3), (4) [22].

(2) $$\;\tau_{Direct}=AT^{-1}\;$$

(3) $$\;\tau_{Raman}=CT^{-m}\;$$

(4) $$\;\tau^{-1}={\tau_{Direct}}^{-1}+{\tau_{Raman}}^{-1}$$

Here, A and C are the coefficients for the direct and Raman processes, respectively, and *m* is the exponent associated with the Raman‐like process. The Raman‐like process encompasses both the conventional Raman relaxation and relaxation via vibrational or local modes [13]. The deviation from the theoretical curve in the low‐temperature region is attributed to the phonon bottleneck effect (τ ∝*T^–^* ^2^) [43], which depends on the measurement conditions. The fitted Raman‐like exponent, *m* = 4.4, is comparable to previously reported values for 1% Cu(II)‐doped Zn(II)‐MOFs with the same host framework (Table S3) [38]. The obtained τ (61 ms at 1.8 K and 1.3 ms at 10 K) are longer than those of a previously reported the Cu(II)‐doped MOF (15 ms at 1.8 K and 0.57 ms at 10 K under 0.4 T). In contrast, they are shorter than those of a 0.2% Co(II)‐doped MOF at lower temperatures (151 ms at 1.8 K under 0.1 T) [37], although longer at higher temperatures (0.29 ms at 6.4 K under 0.1 T). This difference in temperature dependence is reflected in the Raman exponent m. For the Mn(II)‐doped MOF (*m* = 4.4 ± 0.5), whereas for the Co(II)‐doped MOF (*m* = 9), indicating that the magnetic relaxation of Co(II) centers is more sensitive to increasing temperature. From the perspective of spin qubit applications, Mn(II) centers are more favorable to achieve longer magnetic relaxation at a high temperature region.

### 2.2 Spin Qubit Properties

**FIGURE 4:**
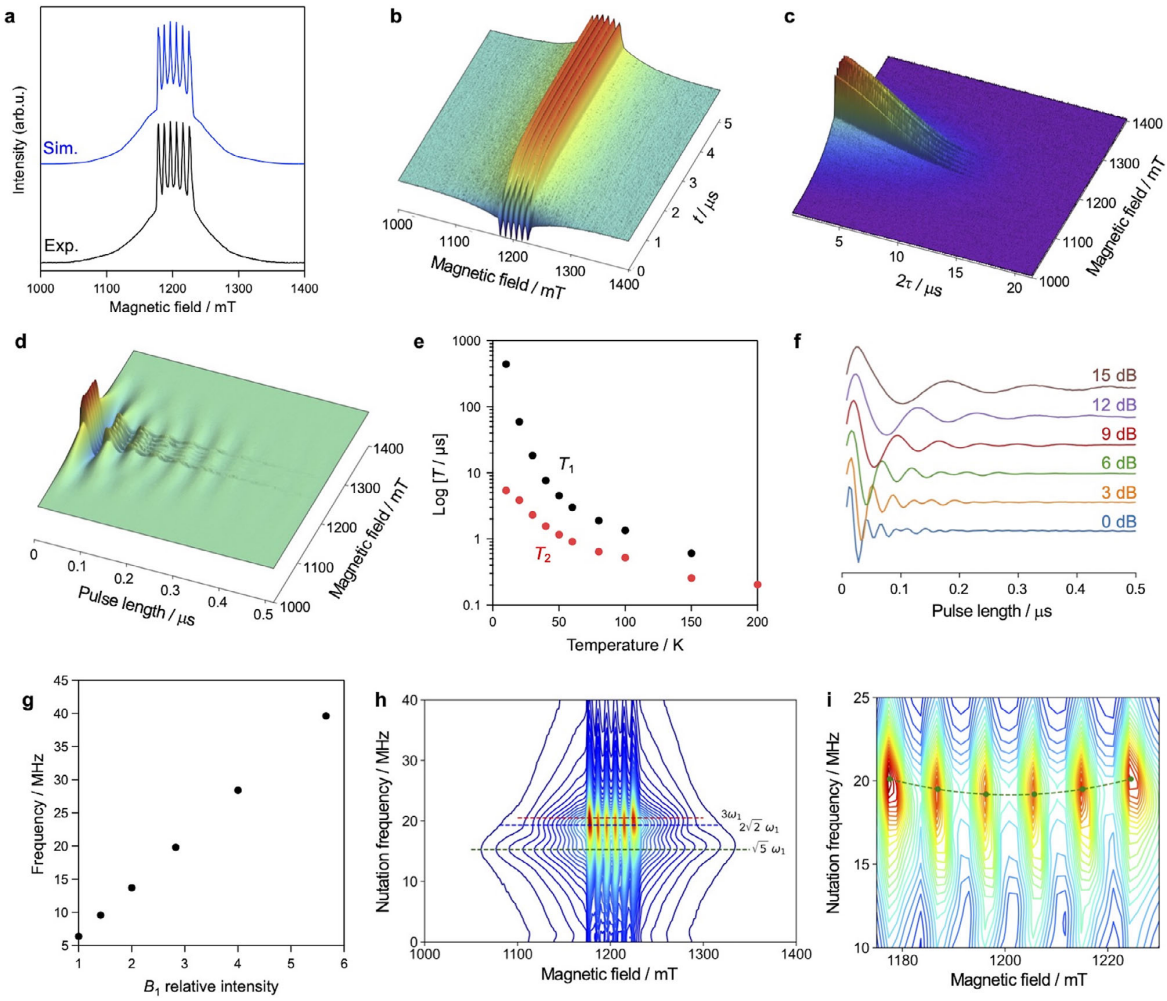
Q‐band pulsed ESR measurements. (a) Hahn echo‐detected field‐swept ESR spectrum of the 0.02% Mn(II)‐doped Zn(II)‐MOF at 10 K under microwave irradiation at 33.6371 GHz, overlaid with a simulated spectrum generated using EasySpin [44] with the parameters: *g*_x_ = 1.997, *g*_y_ = 2.000, *g*_z_ = 2.001; *A*_x_ = *A*_y_ = *A*_z_ = 260 MHz; *D* = 960 MHz, *E* = 160 MHz; and Lorentzian linewidth lwpp = 0.9 mT. 3D plots of decay curves obtained from (b) inversion recovery, (c) Hahn echo, and (d) Rabi nutation measurements at 10 K. (e) Logarithmic plots of *T*_1_ and *T*_2_ relaxation times measured at 1196 mT at 10, 20, 30, 40, 50, 60, 80, 100, 150, and 200 (*T*_2_ only) K. (f) Rabi nutation traces applied 1196.1 mT at 10 K under microwave powers of 0, 3, 6, 9, 12, and 15 dB. (g) Dependence of the Rabi frequency on the relative intensity of the oscillating field *B*_1_. (h) Contour plot of the 2D electron spin transient nutation (2D‐ESTN) signal observed at 10 K under 6 dB microwave power. (i) Magnified view of the 2D‐ESTN signal around the 3ω1 region. Green marks indicate the Winger d matrix elements for *I* = 5/2, $d_{M_{I},-M_{I}}^{I=5/2}\left(12^{\circ }\right)$.

Figure 4 shows the Q‐band pulse‐ESR results of the 0.02% Mn(II)‐doped Zn(II) MOF. The sextet signal appears around 1200 mT derived from the doped Mn(II) centers (*S* = 5/2) with the nuclear coupling (*I* = 5/2). The magnetic parameters were obtained by the simulation using EasySpin [44] to be slightly anisotropic *g* factors (*g*_x_ = 1.997, *g*_y_ = 2.000, *g*_z_ = 2.001) with the nuclear coupling (*A*_x_ = *A*_y_ = *A*_z_ = 260 MHz) (Figure 4a). Additionally, ZFS parameters of *D* = 960 MHz (0.032 cm^−1^) and *E* = 160 MHz (0.0053 cm^−1^) indicates less anisotropy around the doped Mn(II) centers. Magnetic field dependency of the inversion recovery (Figure 4b), Hahn echo decay (Figure 4c), and Rabi nutation (Figure 4d) were measured, respectively. *T*_1_ and *T*_2_ were obtained from fitting using the exponential equations 5 and 6, respectively (Table S4) [14].

(5) $$I=I_{0}\;\left(1-2\exp\left[-\frac{t}{T_{1}}\right]\right)$$

(6) $$I=I_{0}\;\exp\left[-\frac{2\tau }{T_{2}}\right]$$

Here, *I*, *I*_0_, *t* and τ refer to the echo intensity, initial echo intensity, pulse separation between the initial inversion π‐pulse and the two‐pulse sequence for the Hahn‐echo detection, and pulse separation between π/2‐pulse and π‐pulse in the two‐pulse sequence, respectively. *T*_1_ and *T*_2_ indicate minimal dependence on magnetic field strength near the sextet signals (Figure S7). Figure 4e shows the temperature dependence of *T*_1_ and *T*_2_ plotted on a logarithmic scale. At 10 K, *T*_1_ and *T*_2_ are 440 µs and 5.4 µs, respectively, while at 150 K, they decrease to 0.61 µs and 0.26 µs. In comparison, a previously reported 1% Cu(II)‐doped Zn(II)‐MOF exhibits *T*_1_ = 130 µs and *T*_2_ = 0.39 µs at 10 K [38], indicating that the Mn(II) centers in this study have both longer *T*_1_ and *T*_2_, consistent with their magnetic relaxation properties. On the other hand, a 0.1% Cu(II)‐doped Zn(II)‐MOF shows a significantly longer *T*_1_ (2.2 ms at 10 K) [38], attributed to reduced cross‐relaxation due to increased dilution. Cross‐relaxation arises from magnetic dipole–dipole interactions between spin centers. Since Mn(II) has *S* = 5/2, these interactions are expected to be stronger than in the Cu(II) (*S* = 1/2) system, making cross‐relaxation more likely for Mn(II). However, at higher temperatures, this trend reverses: at 50 K, the *T*_1_ of Mn(II) (4.5 µs) is longer than that of Cu(II) (1.9 µs) [38]. Moreover, *T*_2_ of Mn(II) remains consistently longer than that of Cu(II) across the temperature range of 10–50 K. These results suggest that Mn(II) centers are more suitable than Cu(II) as spin qubit candidates operating at elevated temperatures. This difference in temperature dependence likely stems from the nature of the spin‐bearing orbitals [21]. In Cu(II), the unpaired *S* = 1/2 spin resides in the *e*_g_ (d_x_^2^_‐y_^2^) orbital, which strongly couples with vibrational modes through coordination bonding. In contrast, the *S* = 5/2 spin state of Mn(II) arises from occupation of both *e*_g_ and *t*_2g_ orbitals, forming the ^6^S electronic state. Since this state exhibits minimal spin–orbit coupling, its interaction with vibrational modes is expected to be weaker. This feature makes high‐spin Mn(II) spin qubits more advantageous than Cu(II)‐based spin qubits. Additionally, a comparison with previously reported Mn(II)‐doped Zn(II)‐MOFs, i.e., [CH_7_N_2_][Mn^II^_0.001_Zn^II^_0.999_(HCOO)_3_] (*T*_1_ = ∼2.0 µs and *T*_2_ = ∼0.18 µs at 80 K) [34], [C_2_H_8_N_2_][Mn^II^_0.0005_Zn^II^_0.9995_(HCOO)_3_] (*T*_1_ = ∼4.2 µs and *T*_2_ = ∼0.19 µs at 80 K) [35], and [C_4_H_14_N_2_][Mn^II^_0.001_Zn^II^_0.999_(HCOO)_3_] (*T*_2_ = ∼0.037 µs at 200 K) [36], reveals that in the present study (*T*_1_ = 1.9 µs; *T*_2_ = 0.64 µs and 0.20 µs at 200 K), *T*_1_ is shorter while *T*_2_ is longer. The shorter *T*_1_ can be attributed to differences in resonance frequency and magnetic field strength, as this study employed a Q‐band ESR spectrometer (∼34 GHz, ∼1 T), whereas the previous studies were carried out on an X‐band ESR spectrometer (∼9 GHz, ∼0.3 T) [34, 35, 36]. Applying higher magnetic fields (> 0.1 T) induces magnetic relaxation were confirmed (Figure S5). In contrast, the longer *T*_2_ values observed in this study suggest that the Q‐band technique is advantageous for preserving spin coherence.

Figure 4f shows the Rabi nutation behavior of the 0.02% Mn(II)‐doped Zn(II)‐MOF at 10 K, primarily originating from the resonance between the *M*_S_ = ±1/2 sublevels under various microwave power conditions. Fourier transform analysis of the nutation signal (Figure S8) revealed a linear relationship between the *B*_1_ field amplitude (relative microwave intensity) and the nutation frequency (Figure 4g). Additionally, nutation phenomena can be observed until 150 K (Figure S9). Two‐dimensional contour plots of the nutation frequency versus magnetic field (Figure 4h) exhibit resonance features corresponding to transitions at frequencies of 3 ω1, 2√2·ω1, and √5·ω1. These transitions are assigned to Δ*M*_S_ = ±1 transitions between *M*_S_ = ±1/2, ±1/2 and ±3/2, and ±3/2 and ±5/2 sublevels in the sextet spin state (*S* = 5/2) of the Mn(II) centers, respectively. Furthermore, the nutation frequency observed from Mn‐hyperfine transitions with |*M*_S_ = 1/2, *M*_I_ > ↔ |*M’*_S_ = ‐1/2, *M*_I_ > indicated a small nuclear spin sublevel dependence, as shown in Figure 4i. The nuclear spin sublevel dependence is understood as a rotation of the nuclear‐spin quantum axis during the electron spin transition, and has a significant effect on the transition moments as well as the transition probabilities [45]. The contour plot of the observed 2D‐ESTN spectra indicates that the transition intensities depend on the nuclear spin sublevels. For the Mn‐hyperfine transitions with |*M*_S_ = 1/2, *M*_I_ > ↔ |*M’*_S_ = ‐1/2, *M*_I_ >, the signal intensities in the center region have smaller transition probabilities than the signal intensities at both ends. On the other hand, the signals in the center region is stronger in the echo‐detected field‐swept ESR spectrum in Figure 4a. This difference is due to the measurements of the powder sample. The transition probability trend is reasonable for the current Mn‐doped spin system with high‐nuclear spin. The rotation matrix for nuclear spin with *I* = 5/2 is described by Wigner d‐matrix. Analysis using Wigner d‐matrix elements revealed that the quantization axis of the Mn(II) nuclear spin changes by about 12° during the electron spin transition between *M*_S_ = +1/2 and *M*_S_ = ‐1/2 (Figures 4i and S10), indicating the quantization axis of the electron spin is tilted by the existence of the fine structure interaction. The rotation angle itself is a spatially integrated one because of the randomly oriented powder sample. The ESTN property related to the transition probability is attributed to the anisotropic spin system under the fine structure interaction. To the best of our knowledge, such tilting of the quantization axis due to the fine structure interaction is a hidden property, having not been previously reported in spin qubits, and this quantitative observation represents a novel insight into the tunability of anisotropic spin qubit properties.

## 3 Conclusion

This work has demonstrated that dilute Mn(II) centers (∼0.02 mol%) doped into the Zn(II)‐MOF [CH_6_N_3_][Zn(HCOO)_3_] function as high‐spin (*S* = 5/2) spin qubits, exhibiting phase memory times in the microsecond range above 50 K, marking the first such achievement reported for Mn(II)‐based systems. The multiple hydrogen‐bonding networks formed by the organic cations in the MOF lattice help suppress spin–lattice relaxation and prevent phase transitions, thereby extending *T*_2_. The observed resonance between *M*_S_ = ±1/2 sublevels and quantization axis tilting highlights the tunability of spin dynamics. These results underscore the potential of MOFs as versatile platforms for designing high‐spin molecular qubits and advancing the integration of materials chemistry with quantum information science.

## Conflicts of Interest

The authors declare no conflict of interest.

## Supporting information



The authors have cited additional references within the Supporting Information [46, 47].

## Data Availability

The data that support the findings of this study are available from the corresponding author upon reasonable request.
