# Supplementary material for: Magnetic Dynamics and Elongated Coherence of a High‐Spin Mn(II) Qubit Doped Into a Metal‐Organic Framework
Source: Chemistry. 2025 Dec 17;32(5):e02971. doi: 10.1002/chem.202502971 (PMC12865150; doi:10.1002/chem.202502971)
Supplement: Supplementary file 1 — The authors have cited additional references within the Supporting Information [46, 47]. [file CHEM-32-e02971-s001.pdf]

Magnetic Dynamics and Elongated Coherence of a High-Spin Mn(II) Qubit Doped into a Metal-Organic Framework

Shraddha Gupta,[1] Masanori Wakizaka,*[,2] Takehi Yamane,[3] Hisaaki Tanaka,[2] Ryuta Ishikawa,[4] Shinya Takaishi,[1] Kazunobu Sato,*[,3] and Masahiro Yamashita*[,5,1]

1 Department of Chemistry, Graduate School of Science, Tohoku University, 6-3 Aramaki-Aza-Aoba, Aoba-Ku, Sendai 980-8578, Japan.

2 Department of Applied Chemistry and Bioscience, Faculty of Science and Technology, Chitose Institute of Science and Technology, 758-65 Bibi, Chitose 066-8655, Japan.

3 Department of Chemistry, Graduate School of Science, Osaka Metropolitan University, 3-3-138 Sugimoto, Sumiyoshi-Ku, Osaka 558-8585, Japan.

4 Department of Chemistry, Faculty of Science, Fukuoka University, 8-19-1 Nanakuma, Jonan-Ku, Fukuoka 814-0180, Japan.

5 School of Chemical Science and Engineering, Tongji University, Siping Road 1239, Shanghai 200092, PR China.

Email: ma-wakiz@photon.chitose.ac.jp, sato@omu.ac.jp, masahiro.yamashita.c5@tohoku.ac.jp

Contents

1 Methods
2 Figure S1. PXRD of the Mn(II)-doped Zn(II)-MOFs
3 Figure S2. X-band ESR spectra of the Mn(II)-doped Zn(II)-MOF
4 Table S1. CASSCF results of $[Mn^{II}(HCOO)_6]^{4-}$
5 Figure S3. Energy-level diagram of the d orbitals in the $[Mn^{II}(HCOO)_6]^{4-}$ complex
6 Figure S4. Magnetization–field curves for the 0.2% Mn(II)-doped Zn(II)-MOF
7 Figure S5. Field dependence of the AC magnetic susceptibility for the 0.2% Mn(II)-doped Zn(II)-MOF
8 Figure S6. Temperature dependence of the magnetic susceptibility of the 0.2% Mn(II)-doped Zn(II)-MOF
9 Table S2. Summary of χ'' fitting parameters of the 0.2% Mn(II)-doped Zn(II)-MOF
10 Table S3. Summary of fitting parameters for the magnetic relaxation of the 0.2% Mn(II)-doped Zn(II)-MOF
11 Table S4. Summary of fitting parameters of pulse-ESR of the 0.02% Mn(II)-doped Zn(II)-MOF
12 Figure S7. Q-band pulsed ESR measurements of the 0.02% Mn(II)-doped Zn(II)-MOF
13 Figure S8. Fourier transformed nutation spectra of the 0.02% Mn(II)-doped Zn(II)-MOF
14 Figure S9. Rabi nutation and its Fourier transformed nutation spectra of the 0.02% Mn(II)-doped Zn(II)-MOF
15 Figure S10. Wigner d-matrix elements for $I = 5/2$

Methods

Materials. $MnCl_4 \cdot 4H_2O$, formic acid (HCOOH), methanol (MeOH), and *N,N*-dimethylformamide (DMF) were purchased from Fujifilm Wako Pure Chemical Corp. $ZnCl_2$ and Guanidinium carbonate ($[CH_6N_3]_2CO_3$) was purchased from Sigma-Aldrich. $[CH_6N_3][Zn^{II}(HCOO)_3]$ was prepared using the reported procedure.[37]

Synthesis of $[CH_6N_3][Mn^{II}_xZn^{II}_{1-x}(HCOO)_3]$. Formic acid (811 μL, 21.5 mmol) was added to a suspension of $[CH_6N_3]_2CO_3$ (1.55 g, 8.60 mmol) in MeOH (20 mL), yielding a colorless solution. A solution of $ZnCl_2$ (585 mg, 4.30 mmol) and $MnCl_2 \cdot 4H_2O$ (0.20 or 0.02 mol% relative to Zn) in MeOH (20 mL) was added dropwise with stirring at room temperature, immediately producing a precipitate. The mostly white solid was collected by filtration, washed with DMF (3 × 5 mL) and MeOH (3 × 5 mL), and dried under vacuum to afford the MOF in nearly quantitative yield (~100%). Elemental analysis calculated as $C_4H_9N_3O_6Zn$ ($[CH_6N_3][Zn(HCOO)_3]$) (%): C 18.44, H 3.48, N 16.13; found: C 18.52, H 3.59, N 16.14. The Zn/Mn ratio was determined by XRF analysis.

Measurements. The elemental analysis was performed by using J-Science Lab Co. Ltd. JM11 at the Research and Analytical Center for Giant Molecules (Tohoku Univ.). XRF spectroscopy was performed by using a Rigaku NEX-DE under an atmosphere of air. PXRD measurements were carried out with Cu-Kα radiation using a Bruker D8 DISCOVER at room temperature. Magnetic measurements were performed using a magnetic property measurement system (MPMS-XL, Quantum Design) in direct current and alternating current modes at 1.8−11 K. The sample (~100 mg) was filled into a gelatin capsule (Matsuya) which was fixed in a plastic straw. X-band ESR (9.4678 GHz) spectra were measured using a Bruker E-500 spectrometer at room temperature in the solid state.

Pulse-ESR. Pulse-ESR spectra were recorded on a Bruker ELEXSYS E580 at Q-band with a fixed frequency resonator (Bruker EN 5107D2) and an Oxford Instruments CF935 continuous flow helium cryostat. T_1 was measured by the standard inversion recovery sequence (π–t–$\pi/2$–τ–π–τ–echo) with τ = 400 ns, $t_{\pi/2}$ = 100 ns, and $t\pi$ = 200 ns. T_2 were recorded by the Hahn-echo pulse sequence ($\pi/2$–τ–π–τ–echo) with fixed pulse separation τ = 400 ns, $t_{\pi/2}$ = 100 ns, and $t\pi$ = 200 ns. Rabi-nutations were measured with two-pulse sequence which consists of a nutation pulse (t_{nut}) of variable length and π pulse (t_{nut}–τ–π–τ–echo), where the pulse separation τ was fixed to 400 ns. The π pulse length for the echo detection was adjusted depending on the attenuation level.

Quantum Chemical Calculations. Complete active space self-consistent field (CASSCF) and N-electron valence perturbation theory (NEVPT2) calculations were performed using the DKH2-def2-TZVP with the AutoAux basis sets on the program Orca.[46,47] The atomic coordinates were used from crystal structure of $[CH_6N_3][Zn^{II}(HCOO)_3]$.[40] The active space was applied five electrons and five 3d orbitals (CAS(5e,5o)) with 1 sextet, 24 quartet, 75 doublet states on the Mn(II) center.

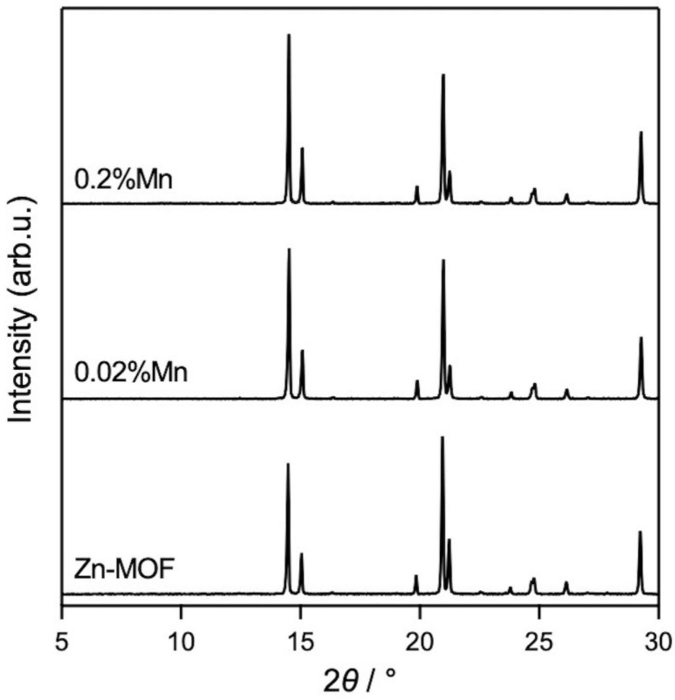

Figure S1. PXRD patterns of the Mn(II)-doped Zn(II)-MOFs together with that of the pristine Zn(II)-MOF, $[CH_6N_3][Zn^{II}(HCOO)_3]$.[39]

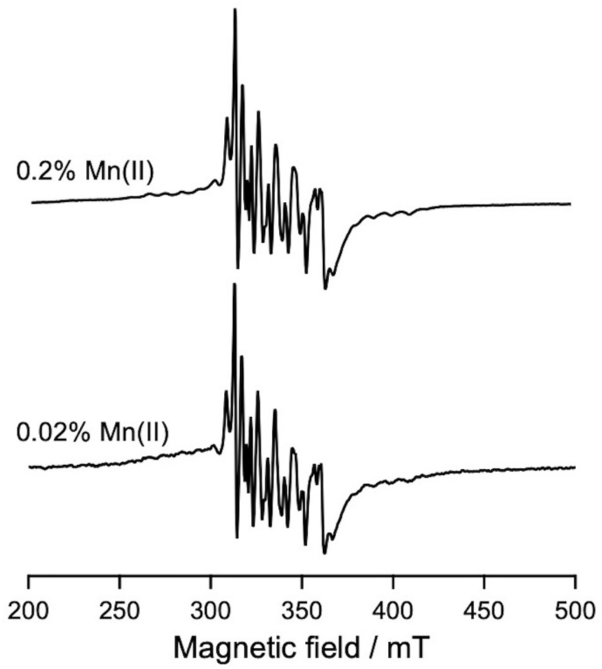

Figure S2. X-band ESR spectra of the 0.2% and 0.02% Mn(II)-doped Zn(II)-MOF at room temperature in the solid state.

Table S1. CASSCF results of $[Mn^{II}(HCOO)_6]^{4-}$

Zero-field splitting parameter	
g_x, g_y, g_z	2.002, 2.002, 2.002
D / cm^{-1}	–0.0388 (–1163 MHz)
E/D	0.326
**Ligand-filed splitting parameter**	
B / cm^{-1}	909.6
C/B	3.876
ζ / cm^{-1}	313.5

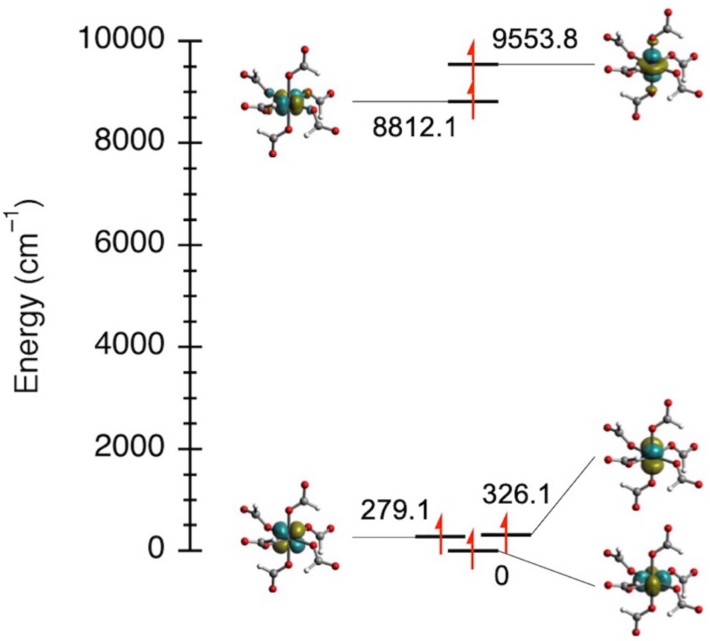

Figure S3. Energy-level diagram of the d orbitals in the $[Mn^{II}(HCOO)_6]^{4-}$ complex.

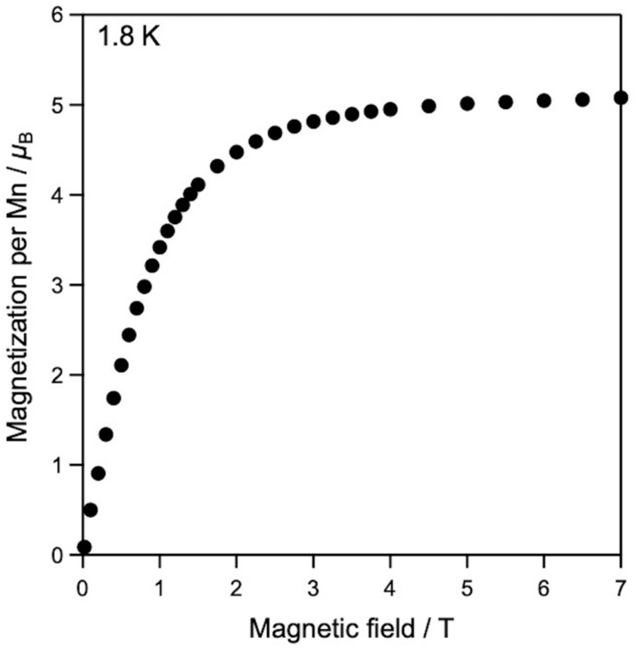

Figure S4. Magnetization–field curves measured at 1.8 K for the 0.2% Mn(II)-doped Zn(II)-MOF.

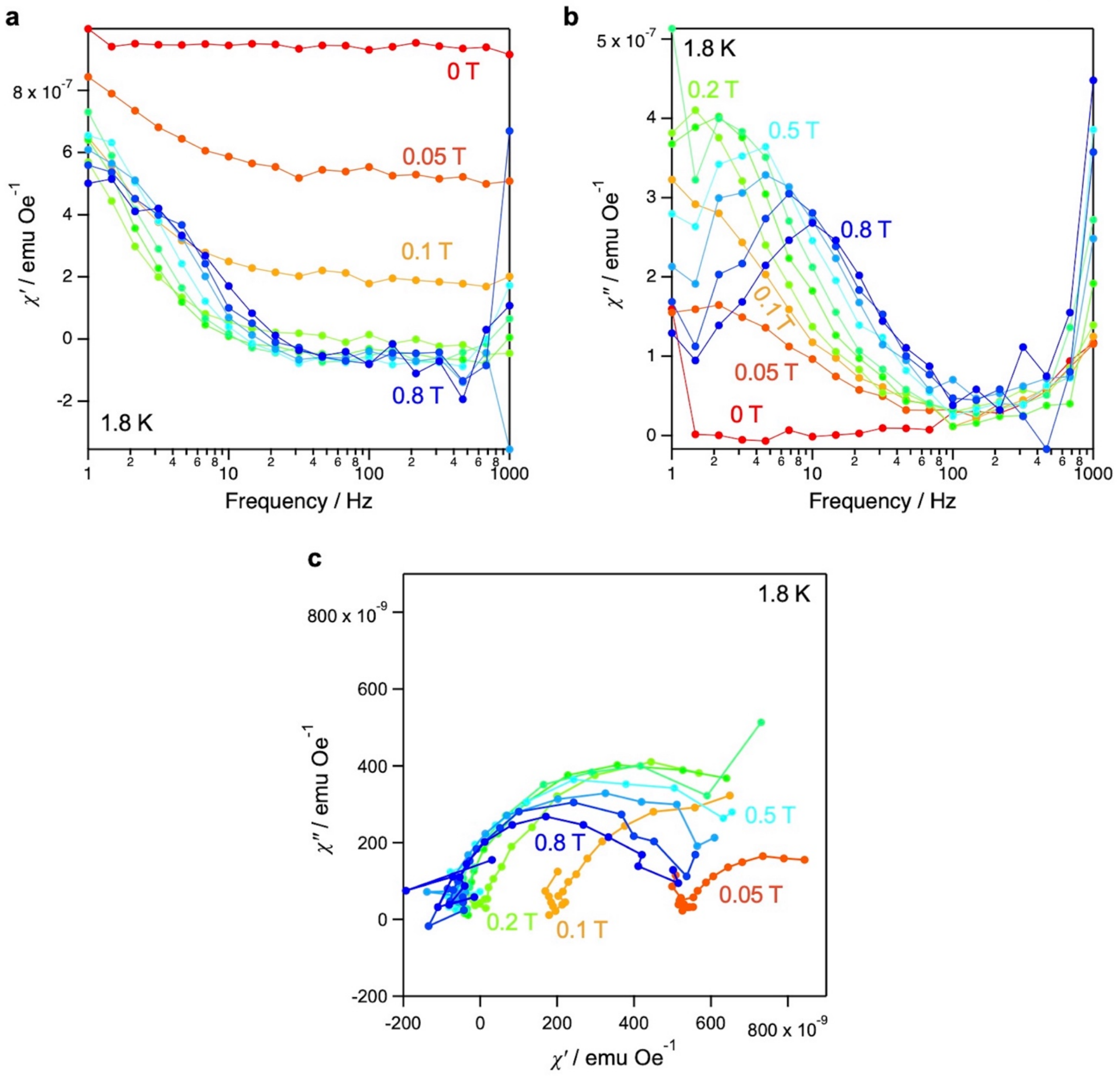

Figure S5. Field dependence of the AC magnetic susceptibility of (a) in-phase, (b) out-of-phase, and (c) Cole-Cole plots for the 0.2% Mn(II)-doped Zn(II)-MOF at 1.8 K under 0, 0.05, 0.1, 0.2, 0.3, 0.4, 0.5, 0.6, 0.7, 0.8 T.

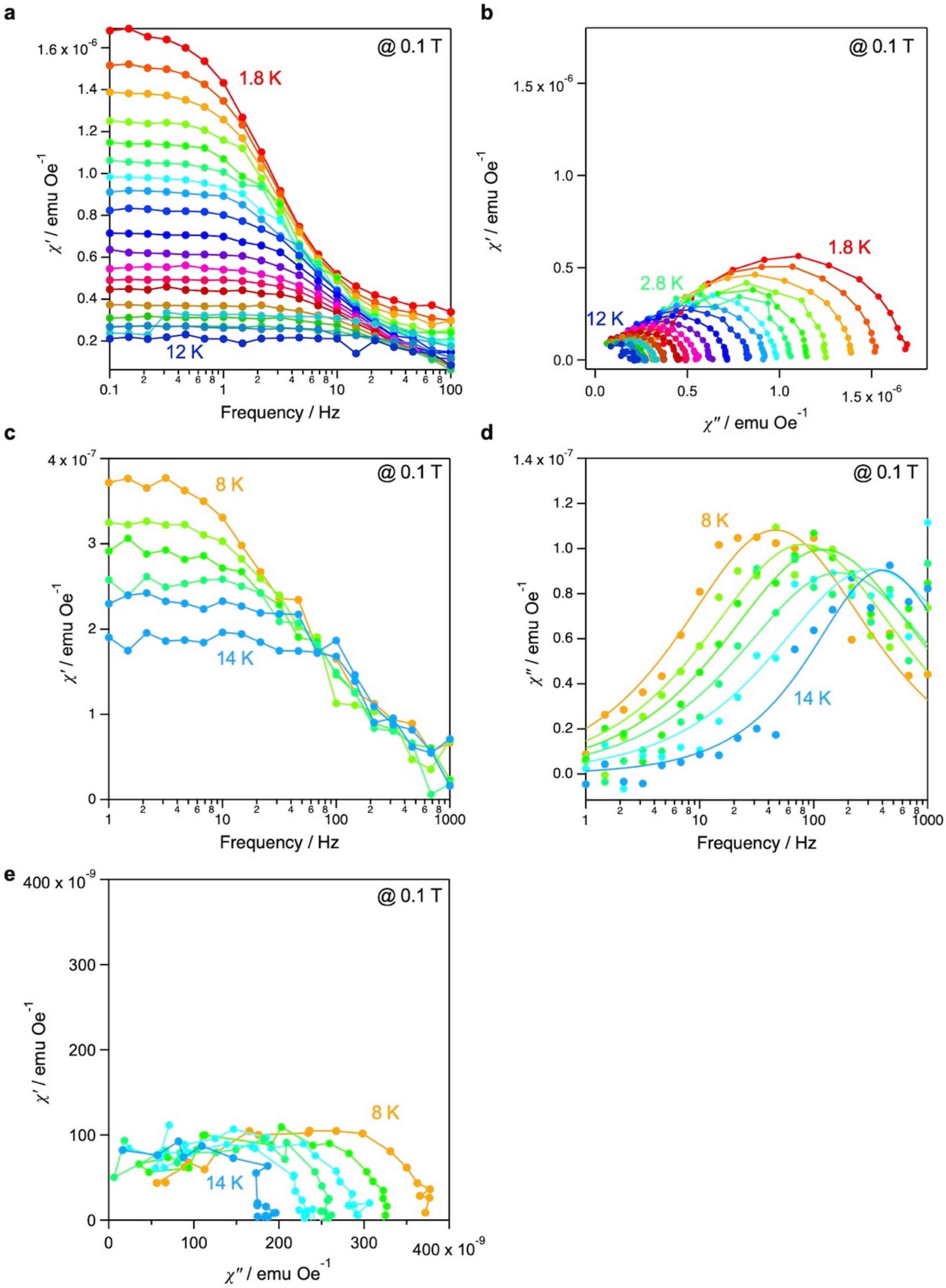

Figure S6. Temperature dependence of the magnetic susceptibility of the 0.2% Mn(II)-doped Zn(II)-MOF at (a, b) 1.8, 2.0, 2.2, 2.4, 2.6, 2.8, 3.0, 3.2, 3.5, 4.0, 4.5, 5.0, 5.5, 6.0, 7.0, 8.0, 9.0, 10, 11, and 12 K over a frequency range of 0.1–100 Hz and (c–e) 8, 9, 10, 11, 12, 14 K over a frequency range of 1–1000 Hz, under 0.1 T.

Table S2. Summary of χ'' fitting parameters of the 0.2% Mn(II)-doped Zn(II)-MOF under 0.1 T

T [K]	$\chi_T - \chi_S$ [emu Oe^{-1}]	τ [s]	α	$\sigma\,\chi_T - \chi_S$ [emu Oe^{-1}]	$\sigma\,\tau$ [s]	$\sigma\,\alpha$
1.8	1.35e−06	0.0609	0.121	1.81e−08	0.00132	0.0111
2.0	1.24e−07	0.0526	0.125	1.51e−08	0.00105	0.0100
2.2	1.14e−06	0.0443	0.133	1.19e−08	0.0007645	0.0086
2.4	1.03e−06	0.0375	0.159	2.88e−08	0.00179	0.0228
2.6	9.60e−07	0.0348	0.155	2.61e−08	0.00161	0.0222
2.8	8.91e−07	0.0278	0.155	2.74e−08	0.00144	0.0250
3.0	8.4e−07	0.0262	0.135	1.93e−08	0.000991	0.0190
3.2	7.95e−07	0.0212	0.167	1.53e−08	0.000697	0.0157
3.5	7.15e−07	0.0179	0.163	9.03e−09	0.000382	0.0104
4.0	6.36e−07	0.0148	0.168	1.07e−08	0.000423	0.0139
4.5	5.73e−07	0.0117	0.189	1.20e−08	0.000423	0.0171
5.0	5.14e−07	0.00957	0.184	1.16e−08	0.000367	0.0184
5.5	4.54e−07	0.00866	0.172	1.03e−08	0.000326	0.0185
6.0	4.4e−07	0.00665	0.232	1.89e−08	0.000540	0.0341
7.0	3.88e−07	0.00521	0.279	1.99e−08	0.000536	0.0396
8.0	3.53e−07	0.00345	0.300	1.39e−08	0.000279	0.0299
9.0	3.45e−07	0.00198	0.322	2.15e−08	0.000259	0.0465
10	3.42e−07	0.00132	0.329	2.33e−08	0.000189	0.0496
11	3.07e−07	0.000986	0.329	3.23e−08	0.000220	0.0752
12	3.12e−07	0.000486	0.330	4.17e−08	0.000144	0.0851
14	2.31e−07	0.000395	0.154	1.79e−08	5e−05	0.0581

Standard deviation (σ)

Table S3. Summary of fitting parameters for the magnetic relaxation of the 0.2% Mn(II)-doped Zn(II)-MOF

A [K s^{-1}]	C [K^m s^{-1}]	m	σA [K s^{-1}]	σC [K^m s^{-1}]	σm
0.0669	45.1	4.37	0.00472	43.5	0.457

Standard deviation (σ)

Table S4. Summary of fitting parameters of pulse-ESR of the 0.02% Mn(II)-doped Zn(II)-MOF

T [K]	T_1 [μs]	σ T_1 [μs]	T_2 [μs]	σ T_2 [μs]
10	442	2.6	5.41	0.060
20	59.3	0.60	3.87	0.028
30	18.4	0.15	2.31	0.011
40	7.64	0.050	1.56	0.0040
50	4.52	0.028	1.16	0.0023
60	3.00	0.017	0.909	0.0014
80	1.89	0.014	0.641	0.0010
100	1.35	0.010	0.520	0.00080
150	0.607	0.028	0.256	0.0012
200	–	–	0.204	0.00010

Standard deviation (σ)

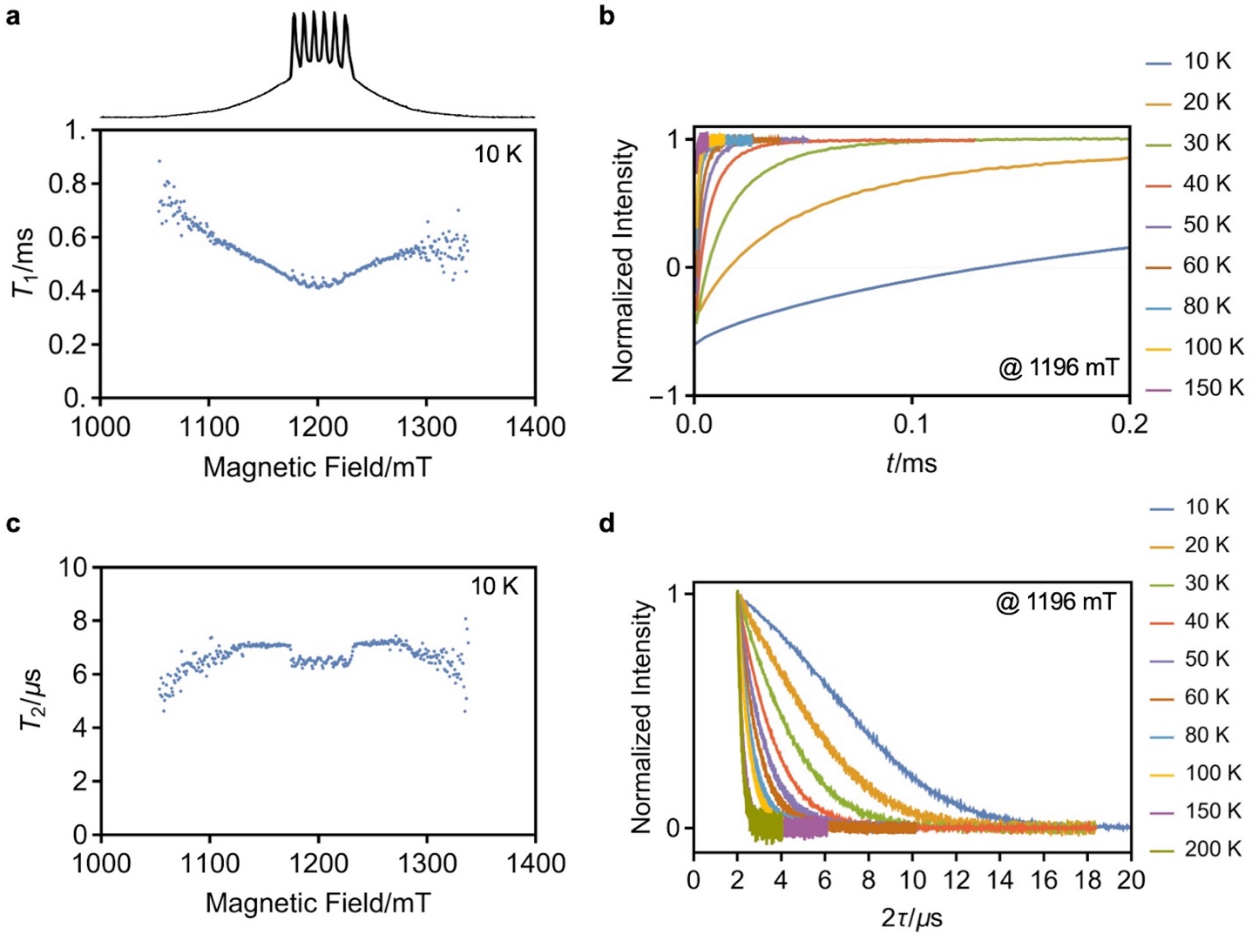

Figure S7. Q-band pulsed ESR measurements of the 0.02% Mn(II)-doped Zn(II)-MOF. (a) Magnetic field dependence of spin–lattice relaxation (T_1) at 10 K, (b) temperature dependence of T_1 at 1196 mT, (c) magnetic field dependence of spin–spin relaxation (T_2) at 10 K, and (d) temperature dependence of T_2 at 1196 mT.

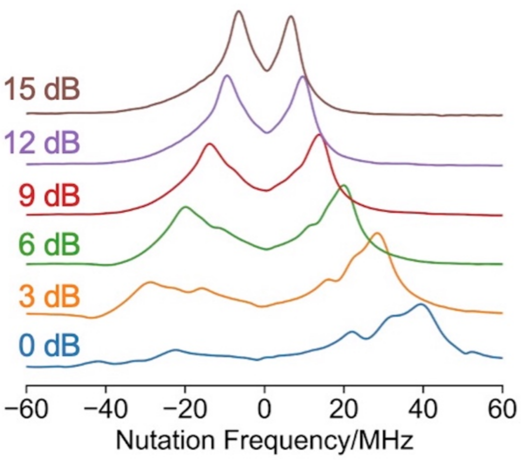

Figure S8. Fourier transformed nutation spectra of the 0.02% Mn(II)-doped Zn(II)-MOF at 10 K and 1196 mT for different microwave powers (0, 3, 6, 9, 12, and 15 dB).

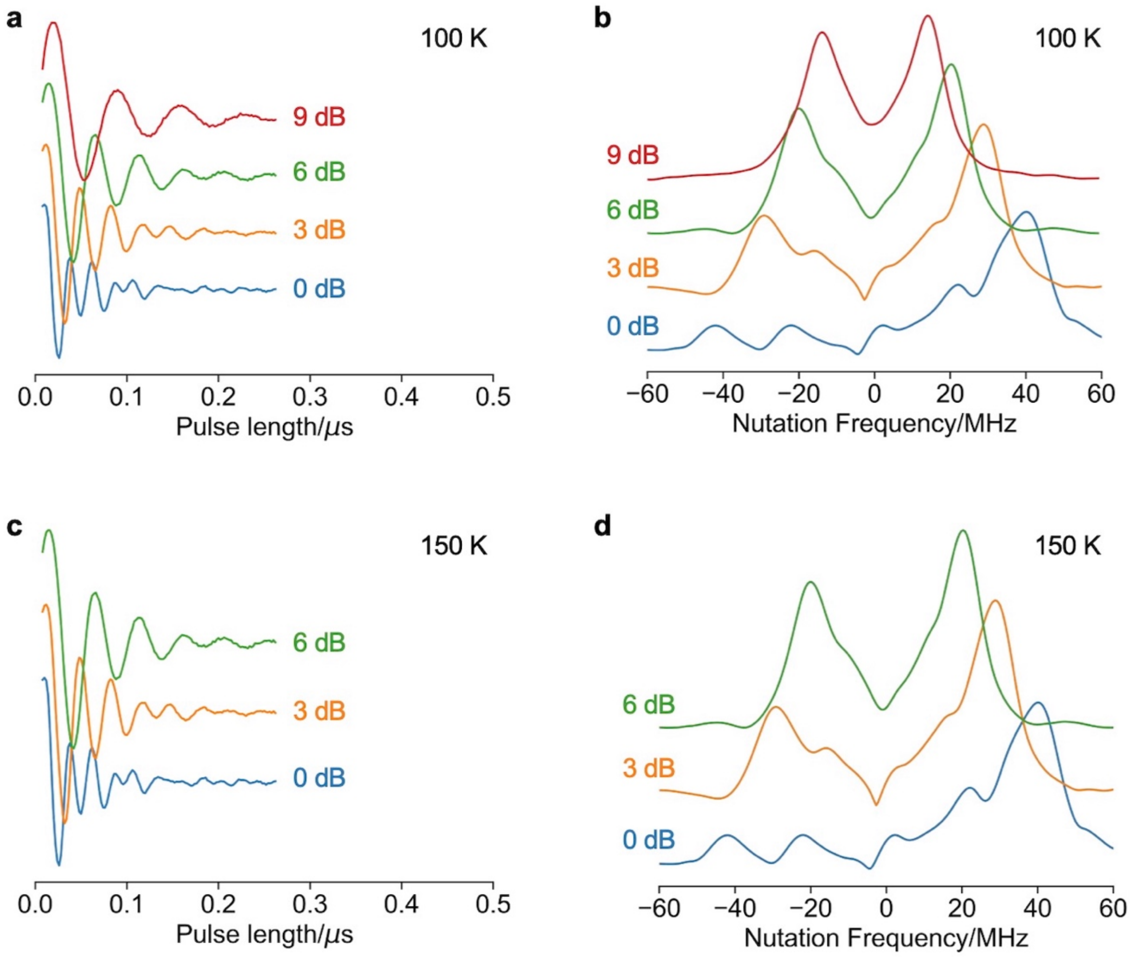

Figure S9. Rabi nutation and its Fourier transformed nutation spectra of the 0.02% Mn(II)-doped Zn(II)-MOF at (a, b) 100 K and (c, d) 150 K at 1196 mT for different microwave power.

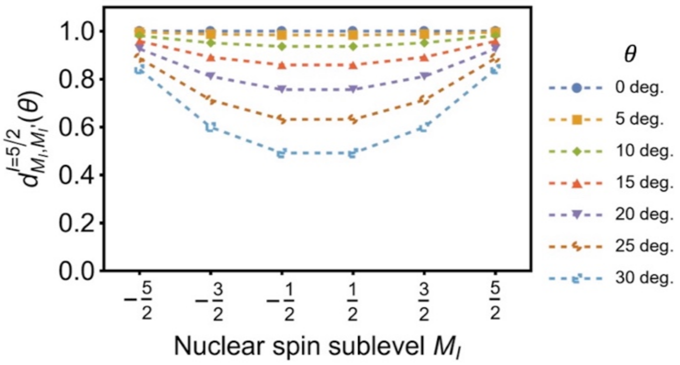

Figure S10. Wigner d-matrix elements for I = 5/2, $d^{I=5/2}_{M_I,M_I'}(\theta)$ ($M_I{}' = M_I$). The elements of $d^{I=5/2}_{M_I,-M_I}(\pi-\theta)$ are equivalent to those of $d^{I=5/2}_{M_I,M_I}(\theta)$.
